# Roles of the *Sec2p* Gene in the Growth and Pathogenicity Regulation of *Aspergillus fumigatus*

**DOI:** 10.3390/jof11010036

**Published:** 2025-01-05

**Authors:** Yuhuan Liu, Shumi Shang, Cong Liu, Yichen Liu, Keyang Xu, Dan He, Li Wang

**Affiliations:** 1 Department of Pathogenobiology, Jilin University Mycology Research Center, Key Laboratory of Zoonosis Research, Ministry of Education, College of Basic Medical Sciences, Jilin University, Changchun 130021, China; yh_liu@jlu.edu.cn (Y.L.); xuky23@mails.jlu.edu.cn (K.X.); 2 Department of Dermatology, The Third Affiliated Hospital of Sun Yat-sen University, Guangzhou 510630, China; liuc_0705@163.com

**Keywords:** *Aspergillus fumigatus*, *Sec2p*, growth, autophagy, cell wall, virulence

## Abstract

*Aspergillus fumigatus* (*A. fumigatus*) is a filamentous fungus that causes invasive aspergillosis in immunocompromised individuals. Regulating fungal growth is crucial for preventing disease development. This study found that deleting the guanine nucleotide exchange factor *Sec2p* gene led to slower *A. fumigatus* growth and reduced the fungal burden and mortality of infected mice. However, the mechanism by which this gene affects *A. fumigatus* growth and pathogenicity remains unclear. Transmission electron microscopy revealed that the vacuoles of the gene knockout strain *ΔSec2p* accumulated more autophagosomes, indicating inhibition of autophagosome degradation. When phenylmethylsulfonyl fluoride was applied to inhibit autophagosome degradation, the *ΔSec2p* strain produced fewer autophagosomes; the *ΔSec2p* autophagy pathway was inhibited, affecting *A. fumigatus*’ nutrient homeostasis and growth. Unlike the wild type, the *ΔSec2p* strain showed strong resistance to cell wall stress. When exposed to caspofungin, *Sec2p* negatively regulated the expression of cell wall integrity (CWI) pathway genes and participated in the cell wall stress response of *A. fumigatus*. Furthermore, this gene positively regulated the autophagy pathway and enhanced CWI pathway gene expression to respond to rapamycin-induced autophagy. In summary, *Sec2p* positively regulated the autophagy pathway; it negatively regulated the CWI pathway during cell wall stress, coordinating the growth and pathogenicity of *A. fumigatus*.

## 1. Introduction

*Aspergillus fumigatus* is a filamentous fungus that can cause invasive aspergillosis in immunocompromised individuals. In recent years, the increasing number of immunosuppressed individuals and global health events such as the COVID-19 pandemic have led to an increasing burden of invasive aspergillosis [1,2,3,4]. Once a person inhales *A. fumigatus* spores, they germinate and establish hyphae in the airways. Hyphae are highly polarized growths that invade tissues and cause destruction. A large amount of published data have shown that gene mutations related to the hyphal morphogenesis process result in a significant reduction in virulence [5]. Therefore, screening for regulatory genes of *A. fumigatus* growth can help identify potential targets for treating fungal infections.

Guanosine nucleotide exchange factor Sec2 (the direct ortholog of Sec2p in *A. fumigatus*) activates the Rab protein Sec4 in *Saccharomyces cerevisiae* [6]. In the presence of Sec2, Sec4 protein is converted into its active GTP-bound form, thereby binding to various effectors [7,8,9]. *Sec2* deletion is lethal in *S*. *cerevisiae* [10], but *AnSEC2* deletion shows only a mild phenotype in *Aspergillus niger* [11], and polarized hypha growth in *Candida albicans* requires Cdc28-Ccn1/Hgc1 kinase phosphorylation of Sec2 [12]. In *S. cerevisiae*, Sec2-mediated Sec4 activation appears to contribute to autophagosome formation [9]. However, the function of Sec2p in *A. fumigatus* is not yet known.

Autophagy is a universal and highly conserved cellular response mechanism that cells initiate in response to nutrient deprivation, hypoxia, and other environmental stresses. Cells degrade cellular organelles through autophagy to provide basic energy for survival [13,14,15,16,17]. In filamentous fungi, autophagy is associated with nutrient balance, secondary metabolism, cell differentiation, and pathogenicity [13,18,19,20]. One of the key components of autophagy is Atg8, which is considered a marker of autophagy [21]. Defects in spore and aerial mycelium growth were observed in mutants of *Aspergillus oryzae* and *Fusarium graminearum* lacking *Atg8* [22,23]. *Atg1*, an essential gene that triggers autophagy, orchestrates intricate signaling pathways to accurately regulate autophagosome formation, and plays a critical role in responding to stress and various biological processes [24]. The absence of *MoAtg1* in *Magnaporthe oryzae* led to defects, reduced spore formation, fewer lipid droplets in spores, malformations, and insufficient adherence pressure [13]. However, the function of autophagy and its regulatory mechanisms in *A. fumigatus* are not fully understood.

In this preliminary study, using *Agrobacterium tumefaciens*-mediated transformation (ATMT) technology [25], transfer DNA (T-DNA) was randomly inserted into the genome of *A. fumigatus* to obtain a mutant with slow growth. The touchdown thermal asymmetric interlaced polymerase chain reaction (TAIL-PCR) [26] product was sequenced to verify that the T-DNA disrupted the AFUA_3G06430 gene, which encodes the GDP/GTP exchange factor Sec2p. To investigate the function of this gene, we constructed a deletion mutant of *Sec2p* to examine its effect on *A. fumigatus* growth, explore its regulatory role in autophagy and cell wall stress, demonstrate its effect on virulence in mice, and characterize new functions of *Sec2p*.

## 2. Materials and Methods

### 2.1. Bacterial Strains, Cells, and Plasmids

The wild-type (WT) *A. fumigatus* strain IFM40808, T-DNA random insertion mutants, *Agrobacterium tumefaciens* strain Agr0, and plasmids pPTRII and pXEH were obtained from the Mycology Research Center at Jilin University (Changchun, China).

### 2.2. Construction of the Sec2p Deletion and Complementation Strains

The *Sec2p* knockout strain was created using homologous recombination via ATMT, with the hygromycin resistance gene replacing the target *Sec2p* gene. To avoid the potential complications of multiple gene copies, the complementation plasmid was constructed from the pPTRII plasmid [27], which provides autonomous replication and does not integrate into the host genome. The *Sec2p* gene in the complementation strain was expressed under the regulation of the *trpC* promoter from *Aspergillus nidulans* [28]. We amplified the *Sec2p* expression sequence from the wild-type *A. fumigatus* strain. The pPTRII plasmid was digested with the restriction endonuclease HindIII, and the resultant three DNA fragments were ligated by using a One Step cloning kit (Vazyme, Nanjing, China). Polyethylene glycol-mediated protoplast transformation was then used to introduce the recombinant plasmid into the *Δsec2p* strain, producing the complementation strain, *sec2pC*. PCR verification confirmed the successful construction of both the *Δsec2p* and *sec2pC* strains (Figure S1). Detailed information on the primers is provided in Supplementary Materials Table S1.

### 2.3. Morphological Examination

Slide cultures were prepared and incubated at 37 °C for 24 h. Samples were then stained with calcofluor white (CFW) (Sigma, St. Louis, MO, USA) and observed under a BX53 fluorescence microscope (Olympus, Tokyo, Japan).

Samples were prepared for transmission electron microscopy (TEM) according to the procedure described by Weichert et al. [29]. Cell wall thickness was determined using ImageJ 1.54g software, with measurements taken at three random locations on the cell wall and averaged to obtain the final thickness value [30].

### 2.4. Animal Infection Models

In this study, 6–8 week male SPF BALB/c mice were used. The mice were divided into four groups: control group, WT group, *Δsec2p* group, and *sec2pC* group. The mouse infection model was developed according to the method described by Zhou et al. [31]. Cyclophosphamide (150 mg/kg) was intraperitoneally injected on days −3, −1, 0, 3, 6, and 9 after infection, and hydrocortisone (40 mg/kg) was subcutaneously injected on day −1 to induce immune suppression. On day 0, the mice were intranasally infected with 30 μL of a conidial suspension (6 × 10^6^ CFU/mL) or saline (control group). Six mice were killed 3 days post-infection, and their tissue was harvested to determine the fungal load and perform histopathological analysis. The survival rate of each group was calculated 14 days post-infection (Figure S2). Survival curves were plotted using GraphPad Prism 9 (GraphPad Software, Santiago, CA, USA) and the data were analyzed using log-rank and Gehan–Breslow weighted Wilcoxon tests.

This study was carried out in accordance with the Guidelines for Care and Use of Laboratory Animals of Jilin University and was approved by the Animal Ethics Committee of Jilin University (protocol code, 2024-332; approval date, 21 July 2024).

### 2.5. Fungal Burden and Histopathology Assays

Fungal burden and histopathology analyses were performed following established protocols [32,33]. Each group of three mouse lungs were homogenized in phosphate-buffered saline (PBS) and subjected to serial ten-fold dilutions for culturing. Fungal burden was assessed by counting colony-forming units (CFUs). Each group of three mouse lungs were paraffin-embedded, sectioned, and stained with hematoxylin and eosin (H&E) or periodic acid–Schiff (PAS) for histopathological analysis.

### 2.6. MDC Staining

The strains were incubated in complete medium for 24 h. Hyphae were washed with sterile distilled water three times, and then transferred to a culture medium containing 2 mM phenylmethylsulfonyl fluoride (PMSF) in the presence or absence of 0.1 μg/mL rapamycin for 4 h. Hyphae were stained with monodansylcadaverine (MDC; Sigma, D4008) at a final concentration of 100 μM in PBS for 10 min in the dark. Following staining, the samples were washed three times with PBS to remove excess dye and then were examined by fluorescence microscopy [34,35]. All the samples were kept on ice in the dark before microscopic observation.

### 2.7. Quantitative Real-Time PCR (qRT-PCR) Analysis

For gene expression analysis, spores (1 × 10^6^ CFU) were incubated in potato dextrose broth (PDB) at 37 °C for 24 h. Mycelia were collected, flash-frozen in liquid nitrogen, and then ground into a powder. Total RNA was extracted using RNAiso Plus (TaKaRa, Osaka, Japan), and cDNA was synthesized using RTIII Super mix (Monad, Shanghai, China). qRT-PCR was conducted using SYBR Green master mix (Monad) on an ABI QuantStudio 3 PCR system (Applied Biosystems, Foster, CA, USA). Gene expression levels related to cell wall stress and autophagy were calculated using the 2^−ΔΔCT^ method, with *A. fumigatus* gene expression normalized to the 18S rRNA housekeeping gene. Primer details are listed in Supplementary Materials Table S2.

### 2.8. Data Analysis

All the experiments were independently replicated at least three times. Statistical analyses were performed using GraphPad Prism version 9 (Dotmatics, San Diego, CA, USA). Comparisons between groups were analyzed by one-way analysis of variance (ANOVA) followed by *t* tests, with statistical significance set at *p* < 0.05. In the cell wall stress response experiment, the inhibition rate was calculated as follows: inhibition rate = (colony diameter without stressors − colony diameter with stressors)/colony diameter without stressors × 100% [36,37].

## 3. Results

### 3.1. Generation and Gene Analysis of the Sec2p Mutant Strain

**Figure 1:**
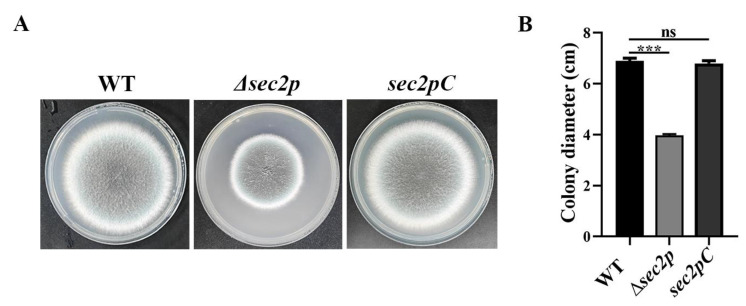
Disruption of *Sec2p* affected the colony morphology of *A. fumigatus*. (**A**) Strains were inoculated on potato dextrose agar (PDA) at 37 °C and grown for 3 days. (**B**) Quantitative data for the results shown in (**A**). ***, *p* < 0.001; ns, not significant.

To investigate the function of the *Sec2p* gene, we constructed an AFUA_3G06430 targeted deletion strain (*Δsec2p*) and a complementation strain (*sec2pC*). The colony morphology was evaluated under 37 °C culture conditions and compared with the WT strain. The diameter of the *Δsec2p* strain cells was significantly reduced (about 42%), while the colony morphology of the *sec2pC* strain showed no significant changes (Figure 1). Together, these results indicated that deleting the *Sec2p* gene inhibited the growth of *A. fumigatus*.

**Figure 2:**
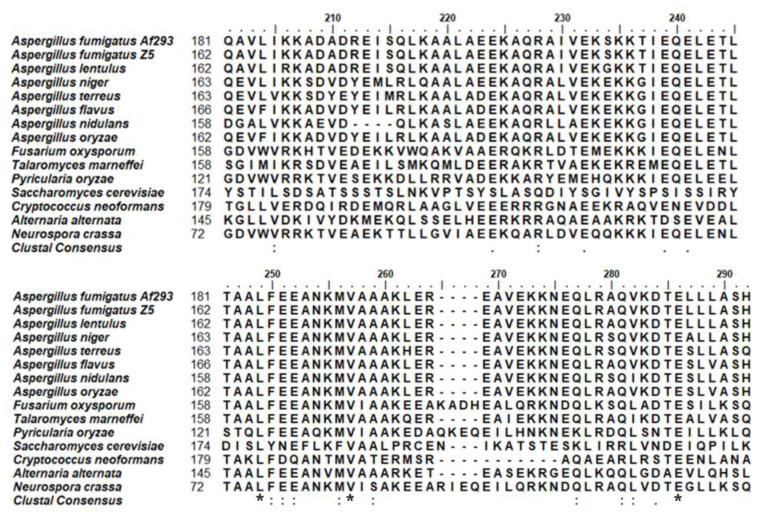
Sec2p is conserved across common pathogenic filamentous fungi. A section of the alignment of the amino acid sequences of Sec2p and its most similar homologs from other fungi, with conserved amino acid residues labeled with *.

The *Sec2p* gene of *A. fumigatus* is located on chromosome 3. The gene consists of 2180 nucleotides with two exons and one intron, encoding a protein comprising 705 amino acids. An amino acid sequence analysis using MEGA indicated that *A. fumigatus* Sec2p was most similar to the homologous proteins of other *Aspergillus* species (79% to 96%), *Fusarium* species (81%), and *Talaromyces marneffei* (78%), but it exhibited low amino acid identity with *Alternaria alternata* (73%), *Neurospora crassa* (67%), *Pyricularia oryzae* (67%), *S. cerevisiae* (38%), and *Cryptococcus neoformans* (less than 11%) (Figure 2).

### 3.2. Phenotypic Analysis of the Sec2p Mutant

**Figure 3:**
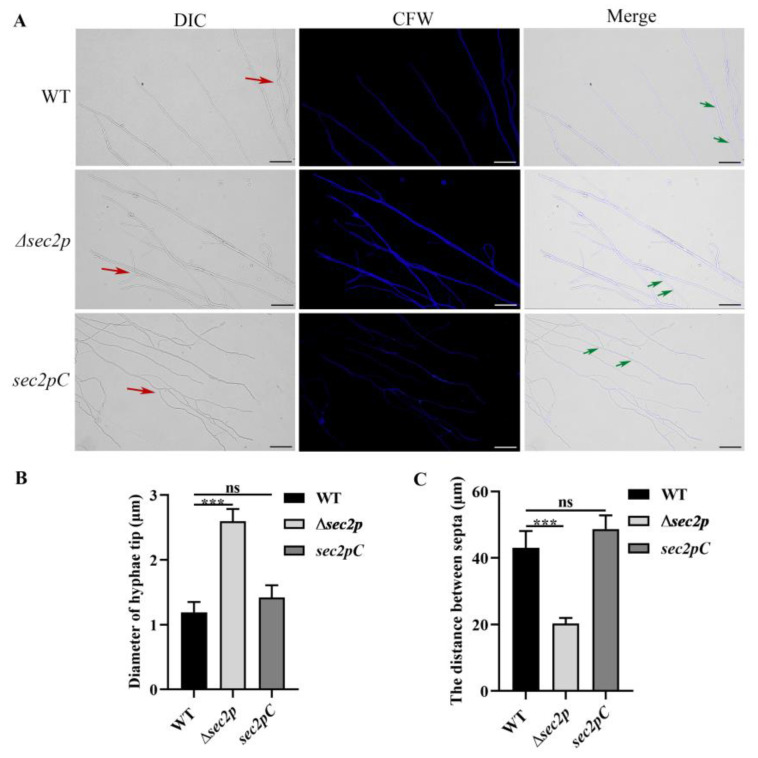
Deletion of *Sec2p* affects the micromorphology of *A. fumigatus*. (**A**) Hyphal morphology after 12 h of growth in PDB. Calcofluor white staining (35 μg/mL) was used for visualization. Red arrows indicate the hyphal branches and green arrows indicate hyphal septa. Bar, 50 μm. (**B**) The diameter of hyphae tip was calculated in triplicate. ***, *p* < 0.001; ns, not significant. (**C**) The distance between septa was calculated in triplicate. ***, *p* < 0.001; ns, not significant.

The effects of *Sec2p* on the morphology of *A. fumigatus* were analyzed using optical microscopy. The results showed that compared with the wild-type strain, the tips of the hyphae of the *Δsec2p* strain were swollen, and the number of hyphal branches and septa increased (Figure 3).

### 3.3. Sec2p Deletion Reduces the Virulence of A. fumigatus

**Figure 4:**
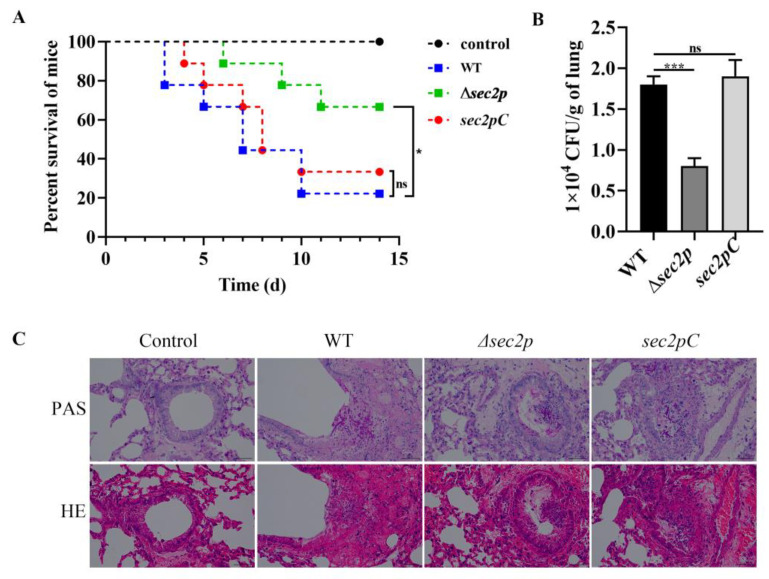
Deletion of *Sec2p* reduces the virulence of *A. fumigatus*. BALB/c mice were challenged with 30 μL of a suspension containing 6 × 10^6^ CFU/mL spores via intranasal instillation. (**A**) Survival rates were monitored over 14 days post-infection. *, *p* < 0.05; ns, not significant. (**B**) Fungal burden in lung lobes from three mice on day 3 post-infection. ***, *p* < 0.001; ns, not significant. (**C**) Histopathological analysis of lung tissue from three mice on day 3 post-infection. Sections were subjected to staining with hematoxylin and eosin (H&E) and periodic acid–Schiff (PAS). Bar, 50 μm.

A mouse model was used to evaluate the impact of *Sec2p* on the virulence of *A. fumigatus*. The survival rates of mice infected with the WT strain and *sec2pC* strain were 22% and 33%, respectively, while the survival rate of mice infected with the *Δsec2p* strain was 67% (Figure 4A). Furthermore, *Sec2p* deletion reduced the fungal burden in the lungs of mice (Figure 4B). A histopathological examination of the lung tissue showed that the bronchial walls were intact in mice infected with the Δsec2p strain, and only a few hyphae were visible. In contrast, the bronchial mucosa was lost, and the airway walls were damaged in mice infected with the WT and *sec2pC* strains, with more inflammatory cells and hyphae visible (Figure 4C). These results suggested that the absence of Sec2p reduced the virulence of *A. fumigatus*.

### 3.4. Sec2p Deletion Affects the Autophagy Pathway in A. fumigatus

**Figure 5:**
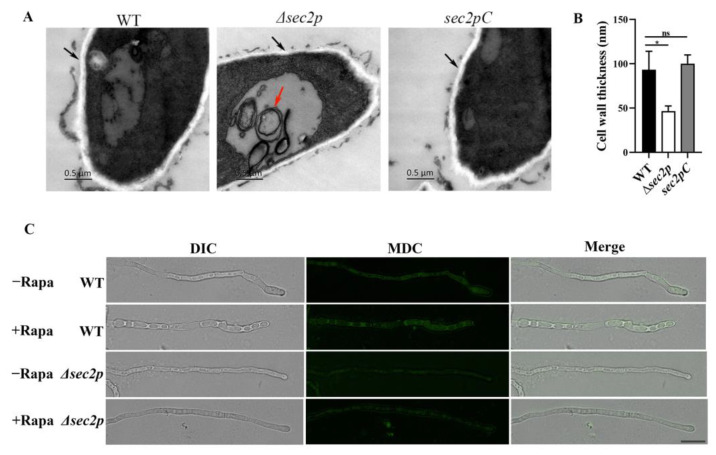
*Sec2p* participates in the autophagic pathway of *A. fumigatus*. (**A**) TEM images of hyphal sections from strains grown in PDB for 24 h at 37 °C. Black arrows indicate the cell wall and red arrows indicate autophagosomes. Bar, 0.5 μm. (**B**) Cell wall thickness was measured at three random locations using ImageJ software, and the average was used as the final measurement. *, *p* < 0.05; ns, not significant. (**C**) Strains were grown in complete medium for 24 h, transferred to a PMSF-containing medium in the presence or absence of rapamycin (Rapa) for 4 h to induce autophagy, stained with 100 μM MDC in the dark for 10 min, and then washed three times with distilled water. Images were captured by fluorescence microscopy. Bar = 50 μm.

Strains were cultured in PDB medium for 24 h, and then the cell ultrastructure was examined using TEM. A large number of autophagosomes were observed in the vacuoles of the *Δsec2p* strain, while no autophagosomes were detected in the vacuoles of the WT or *sec2pC* strains (Figure 5A). Autophagosomes are usually rapidly degraded by hydrolases in the vacuole, making them difficult to visualize under the microscope. This suggests that the absence of *Sec2p* inhibited the degradation of autophagosomes.

Cells were then treated with PMSF to inhibit autophagosome degradation to evaluate the ability of the WT and *Δsec2p* strains to produce autophagosomes. The experimental strains were grown in complete medium and transferred to a PMSF-containing medium in the presence or absence of rapamycin for 4 h to induce autophagy. The cells were then stained with MDC to indicate autophagosomes. The results showed that in the WT strain, the fluorescence of the rapamycin-treated group was stronger than that of the rapamycin-free group, indicating that the WT strain responded to rapamycin induction to produce more autophagosomes. In the *Δsec2p* strain, the mycelium showed weak MDC fluorescence regardless of whether rapamycin was present or not (Figure 5C). This suggests that *Δsec2p* exhibits low autophagy activity under normal conditions and in response to rapamycin induction.

### 3.5. Sec2p Regulates the CWI Pathway to Participate in the Stress Response of A. fumigatus Cells

**Figure 6:**
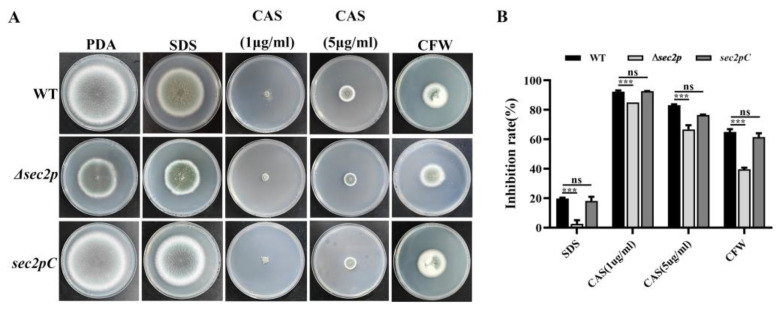
Deletion of *Sec2p* enhances resistance to cell wall-perturbing agents. (**A**) Two microliters of a 1 × 10^6^ CFU/mL spore suspension was inoculated on PDA medium containing 0.02% SDS, 1 μg/mL or 5 μg/mL caspofungin (CAS), or 1 mg/mL CFW, and then incubated at 37 °C for 3 days. (**B**) The relative inhibition rates were calculated in triplicate. ***, *p* < 0.001; ns, not significant.

ImageJ software was used to measure the cell wall thickness of each strain under an electron microscope, and the results showed that the cell wall of the *Δsec2p* hyphae was thinner than that of the WT hyphae (Figure 5A,B), indicating that *Sec2p* may affect cell wall biosynthesis. The sensitivity to various cell wall stressors (chitin synthesis inhibitors sodium dodecyl sulfate (SDS), CFW, and β-1,3-glucan synthase inhibitor caspofungin) was evaluated. All three strains showed growth inhibition on PDA medium supplemented with cell wall stressors. Compared with the WT and *sec2pC* strains, the *Δsec2p* strain was less affected (Figure 6A,B), indicating that the deletion of *Sec2p* increased the resistance of *A. fumigatus* to cell wall perturbagens.

### 3.6. The Effect of Sec2p on the Autophagy Pathway and CWI Pathway-Related Gene Expression Levels in A. fumigatus

#### 3.6.1. Autophagy-Related Gene Expression

**Figure 7:**
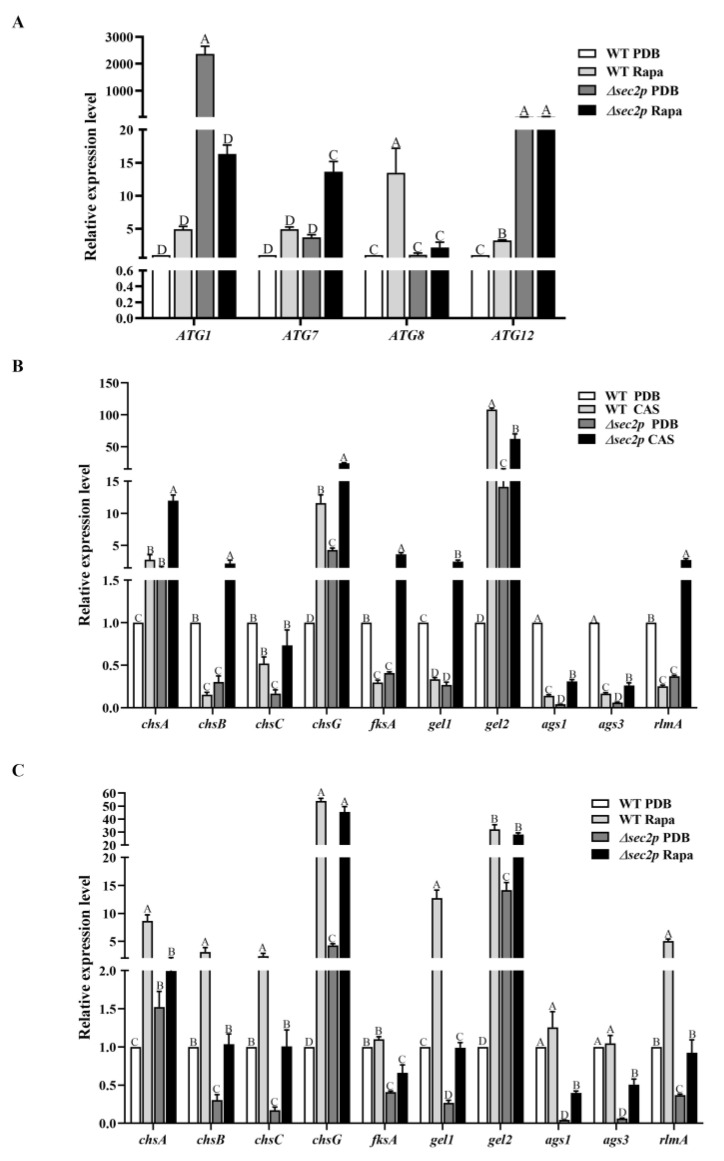
Expression levels of genes related to the autophagy pathway and CWI pathway in *A. fumigatus*. Strains were cultured in PDB medium at 37 °C for 24 h. (**A**) Relative expression levels of autophagy-related genes following rapamycin (Rapa) (0.1 μg/mL) treatment for 4 h. (**B**) Relative expression levels of cell wall synthesis genes following caspofungin (CAS) (1 μg/mL) treatment for 4 h. (**C**) Relative expression levels of cell wall synthesis genes following rapamycin (0.1 μg/mL) treatment for 4 h. Gene expression was measured by qRT-PCR and normalized to the 18S rRNA gene. Letters indicate significant differences (*p* < 0.05).

To characterize the transcriptional regulatory role of *Sec2p* in regulating the autophagy homeostasis of *A. fumigatus*, an analysis of autophagy-related gene expression was conducted. Cells were grown in PDB medium, and the results showed that compared with the WT strain, the expression of the *ATG1* and *ATG12* genes was significantly upregulated in the *Δsec2p* strain, while the expression of *ATG7* and *ATG8* was not significantly different (Figure 7A), indicating that *Sec2p* affected the autophagy homeostasis of *A. fumigatus* under normal conditions. After rapamycin stimulation, the expression level of autophagy-related genes was upregulated in the WT strain, with significant differences observed in *ATG8* and *ATG12*, while no significant differences or decreases were observed in the *Δsec2p* strain (Figure 7A), indicating that *Sec2p* deletion inhibited the rapamycin-induced autophagy pathway of *A. fumigatus*. In summary, *Sec2p* is essential for autophagy in *A. fumigatus*.

#### 3.6.2. The Expression of CWI Pathway Genes

As previously mentioned, *Sec2p* was shown to regulate the integrity of *A. fumigatus* cell walls, which may be reflected in the perturbation of downstream signaling cascades due to cell wall damage. To address this, we evaluated the transcriptome of the CWI transcription factor *rlmA* in *Δsec2p*, which was significantly downregulated. Cells were treated with caspofungin, and *rlmA* expression was upregulated in the *Δsec2p* strain, while downregulated gene expression was observed in the WT strain (Figure 7B), indicating that compared to the WT strain, the CWI pathway in the *Δsec2p* strain exhibited weaker signal transduction without cell wall stressors present, and stronger signal transduction in the presence of cell wall stressors. Further investigation of the transcription levels of the target genes of the CWI pathway, including chitin synthase genes (*chsA*, *chsB*, *chsC*, and *chsG*), α-1,3-glucan synthase genes (*ags1* and *ags3*), the β-1,3-glucan synthase gene (*fksA*), and 1-3-β-glucan glucosyltransferase genes (*gel1* and *gel2*), showed that all the genes were downregulated in the *Δsec2p* strain, except *chsA*, *chsG*, and *gel2*. After caspofungin stimulation, the expression levels of all the genes were upregulated in the *Δsec2p* strain, while in the wild-type strain, expression of all the genes except *chsA*, *chsG*, and *gel2* decreased (Figure 7B), indicating that, in the absence of cell wall stressors and compared to the WT strain, the *Δsec2p* strain exhibited reduced cell wall synthesis. In the presence of cell wall stressors, compared to the WT strain, the *Δsec2p* strain significantly increased cell wall synthesis, which was consistent with the phenotypic change of enhanced resistance to cell wall stressors of the *Δsec2p* strain. These results showed that the *Sec2p* gene negatively regulated the CWI pathway under cell wall stress conditions, inhibiting cell wall synthesis.

#### 3.6.3. Under Rapamycin Induction, Sec2p Positively Regulates the Autophagy Pathway to Enhance the Expression of Genes in the CWI Pathway

To investigate the effect of the autophagy pathway on the CWI pathway, strains were treated with rapamycin, and the transcription level of CWI pathway genes was analyzed. The results showed that with rapamycin stimulation, the expression of the *rlmA*, *chsA*, *chsB*, *chsC*, *chsG*, *gel1*, and *gel2* genes was significantly upregulated in the WT strain (Figure 7C). The expression of these genes in the *Δsec2p* strain was only slightly upregulated. This indicated that rapamycin enhanced the expression of CWI pathway genes, while the absence of *Sec2p* negatively regulated this process. As mentioned earlier, we found that *Sec2p* positively regulated rapamycin-induced autophagy in *A. fumigatus* (Figure 5A,C and Figure 7A). It was reported that autophagy activates the CWI pathway in fungi [38,39], and therefore it was speculated that under autophagy-inducing conditions, *Sec2p* positively regulated the autophagy pathway to enhance the expression of CWI pathway genes.

## 4. Discussion

Research has shown that virulence is related to some extent to the growth of microorganisms [40]. Autophagy pathways degrade waste organelles and other substances, improving cell survival [14]. The CWI signaling pathway is crucial for maintaining the cell shape, resisting cellular stress from the outside world, and regulating cell growth [14]. In this study, it was found that disrupting the *Sec2p* gene in *A. fumigatus* led to colony growth defects (Figure 1) and inhibited the autophagy pathway (Figure 5A,C and Figure 7A). Regulating the cell wall integrity pathway can enhance resistance to cell wall stressors (Figure 6). More importantly, the absence of *Sec2p* reduced the pathogenicity and infection of *A. fumigatus* (Figure 4).

Autophagy is a highly conserved metabolic degradation pathway that uses autophagosomes to package cellular material, fuses the autophagosomes with lysosomes to degrade the cellular material, and then releases and recycles the cellular material to support cellular metabolism and maintain nutrient homeostasis [41,42]. Using TEM, we found that the absence of *Sec2p* inhibited the degradation of autophagosomes in *A. fumigatus* (Figure 5A). *Sec2p* deletion also inhibited autophagosome formation, as shown by MDC staining (Figure 5C), thus indicating that *Sec2p* deletion inhibited the autophagy pathway. Internal cytosol and organelle recycling through autophagy is crucial for providing nutrient transport along the hyphae [43,44]. In *S. cerevisiae*, autophagosome formation depends on Sec2-mediated activation of Sec4 [9]. Therefore, it was hypothesized that *Sec2p* deletion in *A. fumigatus* may inhibit Sec4 activation, thereby inhibiting the autophagy pathway, reducing hyphal nutrient transport, and ultimately resulting in slower growth (Figure 1). In fungal pathogens, successful infection may depend on autophagy-mediated recovery of macromolecules to support host cellular activity under nutrient-limiting conditions [45]. We found that the mortality rate, fungal load, and severity of lesions in mice infected with the *Δsec2p* strain were reduced (Figure 4), and autophagy homeostasis was disrupted, suggesting that *Sec2p* deletion reduced the virulence of *A. fumigatus* by disrupting autophagy.

In pathogenic fungi, *Sec2p* is crucial for vesicle transport and hyphal growth [46,47], but its other specific functions remain unclear. The fungal cell wall is critical in pathogenic fungi because it is responsible for the survival, adaptation, and signal transduction of the pathogen during the stress conditions of infection [48,49]. The CWI pathway is the main signal transduction pathway controlling the response of *S. cerevisiae* to environmental stress and the synthesis of cell wall components [50]. The CWI pathway was found to be critical for the growth and pathogenicity of *M. oryzae* [51]. Although the CWI pathway is relatively conserved among different fungi, the biological functions of the CWI pathway may differ among fungi. We found that the *Δsec2p* strain had thinner cell walls in the nutrient state (Figure 5A,B), but had stronger resistance to cell wall stressors (Figure 6A,B). At the transcriptional level, *Sec2p* interfered with the expression of the CWI pathway genes in *A. fumigatus* under stress conditions, and therefore the CWI target genes, such as genes encoding chitin synthase and β-1,3-glucan synthase, were also misregulated (Figure 7B).

As a key process in response to environmental stress, autophagy and its connection to CWI signals was previously studied in *S. cerevisiae* and *M. oryzae* [38,52,53,54]. In *M. oryzae*, endoplasmic reticulum stress induced by protein synthesis abnormalities activated MoAtg1-dependent MoMkk1 phosphorylation, thereby enhancing CWI signal transduction and promoting infection [38]. This suggested an important relationship between the CWI signal transduction pathway and autophagy. In this study, CWI signal transduction was enhanced in the WT strain under rapamycin-induced autophagy, while the absence of *Sec2p* negatively regulated this process (Figure 7). This indicated that in the presence of rapamycin, *A. fumigatus Sec2p* positively regulated the autophagy pathway to enhance CWI pathway gene expression. However, the additional functions and interacting proteins of the Sec2p gene in *A. fumigatus* remain to be elucidated and warrant further investigation.

In conclusion, we showed that *Sec2p* plays an important role in regulating the growth and virulence of *A. fumigatus*, probably by regulating the autophagy pathway. Furthermore, it affected the sensitivity of *A. fumigatus* to cell wall disruptors and the cell wall thickness by regulating the expression levels of CWI pathway genes, thus coordinating the growth and virulence of *A. fumigatus*. Furthermore, our study showed that *Sec2p* positively regulated the autophagy pathway to enhance the expression of CWI pathway genes in the presence of autophagy inducers. In summary, a new function of *Sec2p* was characterized, which may become a new target for the prevention and treatment of *A. fumigatus* infections.

## Data Availability

The data that support the findings of this study are available from the corresponding author upon reasonable request.

## Supplementary Materials

The following supporting information can be downloaded at https://www.mdpi.com/article/10.3390/jof11010036/s1: Table S1: Primers used for the study of deletion and complementary strains; Table S2: Primers used in study for qRT-PCR.
