# Supplementary material for: Roles of the Sec2p Gene in the Growth and Pathogenicity Regulation of Aspergillus fumigatus
Source: J Fungi (Basel). 2025 Jan 5;11(1):36. doi: 10.3390/jof11010036 (PMC11767236; doi:10.3390/jof11010036)
Supplement: Supplementary file 1 [file jof-11-00036-s001.zip › Table S1.pdf]

Supplementary Table S1. Primers used for the study of deletion and complementary strains.

Primer Name	Nucleotide Sequence(5' to 3')	Purpose
P1<br>P2	CGGAATTCCGCGTGGGACGCTGACTAATG<br>GGGGTACCCCACAGGCGATAACGGACTTG	Amplify sequence upstream of *Sec2p*
P3<br>P4	CGGGATCCCGCGCACCACCATTTCTACAGT<br>CCCAAGCTTGGGACTCATCTTTCCCCTTCTCA	Amplify sequence downstream of *Sec2p*
P5<br>P6	TTCCGGTCCCAGGTCACTTT<br>GCGGCGACCATCTATTCCAG	Amplify sequence of *Sec2p*
P7<br>P8	CGCCCAAGCTGCATCATCGAA<br>CGACAGCGTCTCCGACCTGA	Amplify sequence of hph
P9<br>P10	GACCATGATTACGCCAAGCTTCAG AAGATGATATTGAAGGAGCATTT<br>CCGATTGCATATCGATATCGATGCTTCGGTAGA	Amplify sequence of Trpc promoter
P11<br>P12	CGATATCGATTTCCGGTCCCAGGTCACTTTT<br>GACCTGCAGGCATGCAAGCTTGCGGCGACCATCTATTCCAGTCC	Amplify sequence of *Sec2p* (for complementation)
