# Supplementary material for: Roles of the Sec2p Gene in the Growth and Pathogenicity Regulation of Aspergillus fumigatus
Source: J Fungi (Basel). 2025 Jan 5;11(1):36. doi: 10.3390/jof11010036 (PMC11767236; doi:10.3390/jof11010036)
Supplement: Supplementary file 1 [file jof-11-00036-s001.zip › Table S2.pdf]

Supplementary Table S2. Primers used in study for qRT-PCR.

Primer Name	Nucleotide Sequence(5’ to 3’)	Purpose
ChsAf1	GGATGATGACAGGGCCACAA	*chsA*
ChsAr1	ATCAAACGCCTCGGAACTGT	
ChsBf1	GAAACACACTTCACCCGCAC	*chsB*
ChsBr1	AGTGCGTTCAGAGTCCTTGG	
ChsCf1	CGGATGTCGGGATACAGTGG	*chsC*
ChsCr1	TCAACGCTGAGTACCGAACC	
ChsGf1	ATGTGCCACCAGTCGAGAAG	*chsG*
ChsGr1	AGCAGAATTCGTGAAGCCGA	
FksAf1	CACTTCTTTCCTTGCACGCC	*fksA*
FksAr1	ACGTTCTCTGGGTTCTGCTG	
Gel1f1	TGCGTCAGTACATCCGTAGC	*gel1*
Gel1r1	GATCGCACCAGGAGTAGTCG	
Gel2f1	CACCAAGGACAAGGACCCTC	*gel2*
Gel2r1	CTGCGTTGAAGATGGATGCG	
Ags1f1	TCATTGGCCTCGCCCATTTC	*ags1*
Ags1r1	TGACATAAGCCTGCTGCGTG	
Ags3f1	ACCCCTCAGATACTGGTGCC	*ags3*
Ags3r1	CACCGTCCAACGAAGACGAG	
RlmAf1	CATCCCATGCCTCAACCAGT	*rlmA*
RlmAr1	CTTGTTCGGGCAAGAAAGGC	
ATG1f1	CACCTCAAGCTACTGTCGGG	*ATG1*
ATG1r1	CCAGGGGTGGAGAAAATCCC	
ATG7f1	TCGCGACATCGATAAGCCTC	*ATG7*
ATG7r1	ATGAAGAGCAGGAAACGCGA	
ATG8f1	ACCTGCGGACCTTACAGTTG	*ATG8*
ATG8r1	GTCACCGAAAGTGTTCTCGC	
ATG12f1	GTGACAGTTCGCTTTCAGCC	*ATG12*
ATG12r1	GAGCAAAGACGCTGTTGACG	
