# Supplementary figures and images for: Roles of the Sec2p Gene in the Growth and Pathogenicity Regulation of Aspergillus fumigatus
Source: J Fungi (Basel). 2025 Jan 5;11(1):36. doi: 10.3390/jof11010036 (PMC11767236; doi:10.3390/jof11010036)

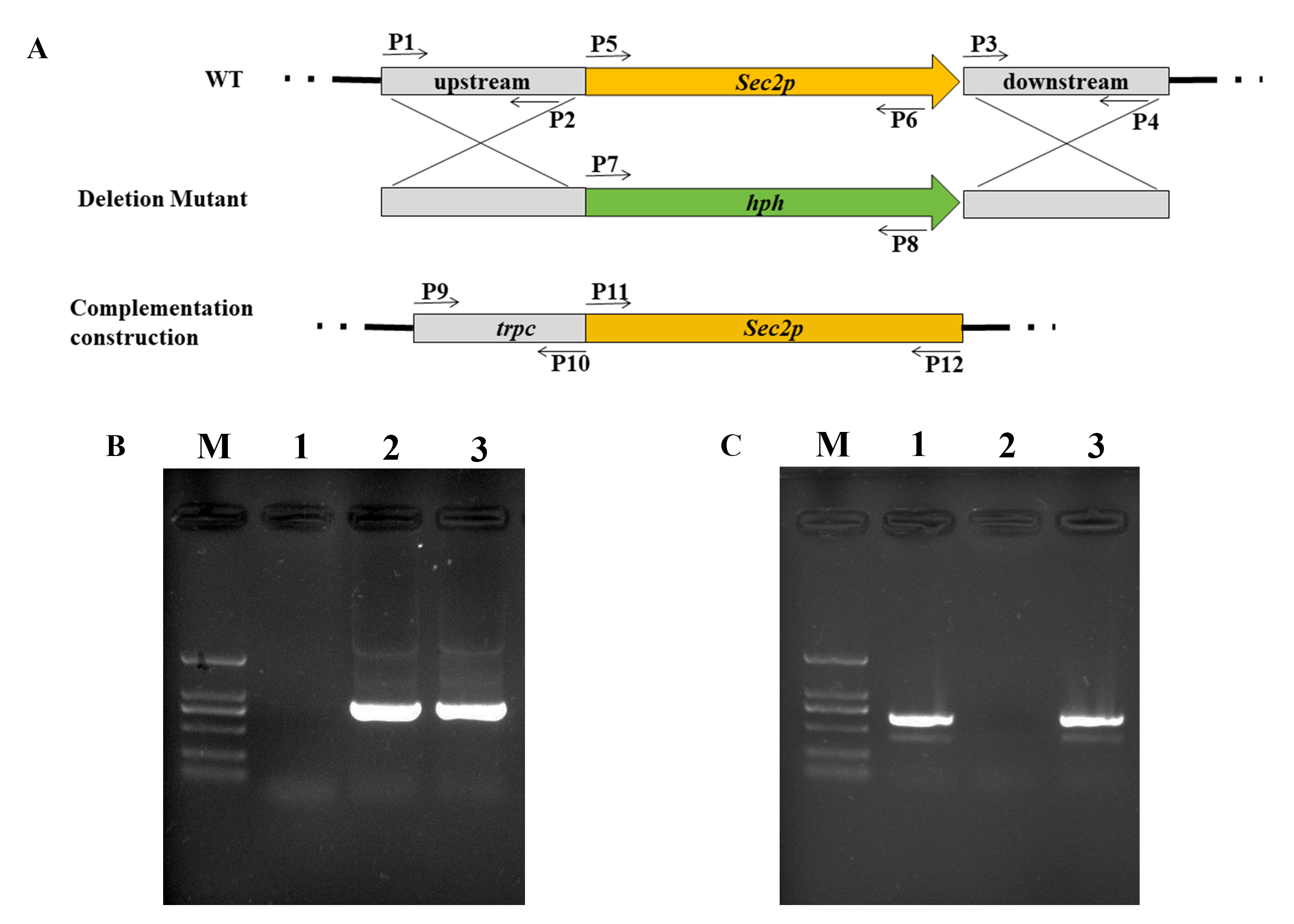

Supplement: Supplementary file 1 [file jof-11-00036-s001.zip › Figure S1.png]

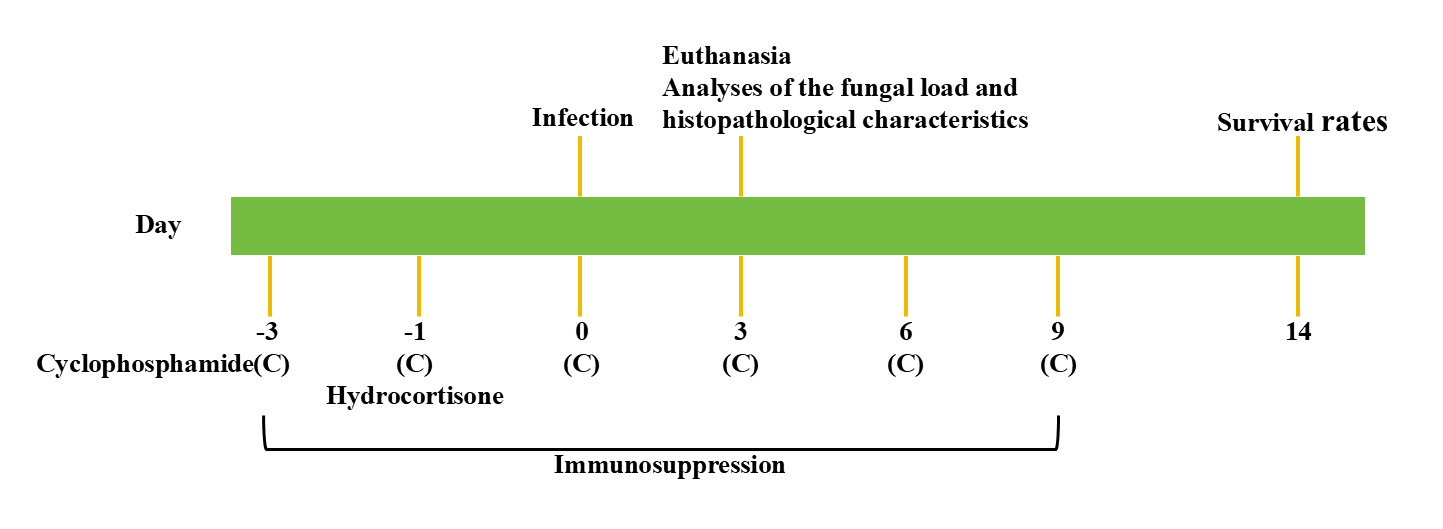

Supplement: Supplementary file 1 [file jof-11-00036-s001.zip › Figure S2.tif]
